# *In-situ* formed thermosensitive hydrogel amplifies statin-mediated immune checkpoint blockade for coordinated tumor chemo-immunotherapy

**DOI:** 10.3389/fphar.2023.1154392

**Published:** 2023-05-09

**Authors:** Zefan Liu, Yajun Zhang, Jinyu Huang, Yan Wang, Xin Kang

**Affiliations:** Department of General Surgery, First People’s Hospital of Shuangliu District, Chengdu, China

**Keywords:** small molecule, immune checkpoint inhibitor, immunogenic cell death, thermosensitive hydrogel, chemo-immunotherapy

## Abstract

Small molecule drugs are the next-generation of immune checkpoint inhibitors (ICIs), but their *in vivo* therapeutic outcomes remain unsatisfactory for a long time. Herein, we proposed a combinatory regimen that delivered a small molecule ICI and an immunogenic cell death inducer in an *in-situ* formed hydrogel scaffold based on thermosensitive materials (Pluronic F127). This platform increased the tumor retention of administrated small molecules, creating more opportunities for the interaction between drugs and tumor cells. We found that atorvastatin (ATO) effectively downregulated the expression of programmed death ligand 1 (PD-L1) and reversed compensative PD-L1 upregulation after cyclophosphamide (CTX) chemotherapy on CT26 colon tumors. CTX not only killed tumor cells to reduce the tumor burden, but also release damage-associated molecular patterns (DAMPs) to stimulate T cell immunity, therefore amplifying statin-mediated immunotherapy. The platform reported in this study might be promising to overcome the limitation of small molecule ICIs with short retention time and potentiate tumor chemo-immunotherapy.

## 1 Introduction

At present, immune checkpoint blockade (ICB) therapy has been well studied and rapidly developed in cancer therapies (Topalian et al., 2016; Wei et al., 2018; Kalbasi and Ribas, 2020). Immune checkpoint proteins deactivate and deplete cytotoxic T lymphocytes (CTLs) with tumor progression (Cogdill et al., 2017). Immune checkpoint inhibitors (ICIs) can abrogate this function and restore anti-tumor immune response (Darvin et al., 2018). Monoclonal antibodies that target checkpoints, such as anti-programmed death 1 antibodies (αPD-1), anti-programmed death ligand 1 antibodies (αPD-L1), and anti-cytotoxic T lymphocyte-associated antigen 4 antibodies (αCTLA-4), have shown profound therapeutic effects in clinical practice, but their disadvantages, including poor tumor permeability, instability, high costs, and immune-related adverse events (irAEs) have also emerged (Webster, 2014; Li et al., 2019; Martins et al., 2019). Small molecule drugs are the new direction in ICB drug development. Exploring the ICB function of old compounds and discovering new chemical entities (NCE) are the two main tactics in this field (Sasikumar and Ramachandra, 2018; Osipov et al., 2019; Park et al., 2021; Wu et al., 2021; Wu et al., 2022).

Statins are drugs that reduce cholesterol by selectively inhibiting 3-hydroxy-3-methylglutaryl coenzyme A (HMG-CoA) reductase (Wong et al., 2002; Palmer et al., 2013). These drugs have been widely used to treat cardiovascular diseases for a longtime, and their anti-tumor activity has only been found recently (Jakobisiak and Golab, 2003; Chen et al., 2020; Wang et al., 2022). It is reported that statins downregulate the expression of PD-L1 in melanoma and lung cancer cells through AKT and *β*-catenin signaling pathway, and inhibit the secretion of PD-L1-rich extracellular vehicles (EVs) from cancer cells (Lim et al., 2021; Nam et al., 2021; Choe et al., 2022). All these evidences indicate that statins can become promising small-molecule ICIs. For the treatment of cardiovascular diseases, statins are suitable for systemic administration (Taylor et al., 2011). But this drug delivery route may not applicable for tumor elimination. Small molecules that circulate in blood will be rapidly metabolized by liver and cleared by kidney (Farokhzad and Langer, 2009; Xiang et al., 2020). Furthermore, free drugs could hardly penetrate into deep tumor tissues (Waite and Roth, 2012; He et al., 2020). These problems would seriously hamper the outcome of statin-based ICB therapy. Therefore, it is urgent to develop a method for the specific delivery of small-molecule ICIs into tumors.

Hydrogel is a three-dimensional network formed by cross-linked hydrophilic polymers (Cully, 2015; Li and Mooney, 2016). It is widely used in drug delivery because of several advantages such as high drug capacity, tissue adhesiveness, low immunogenicity, and controllable drug release behaviors (Hoare and Kohane, 2008). Injectable hydrogel is especially suitable for cancer therapy than other drug delivery systems (DDSs) (Norouzi et al., 2016). Because it can be directly injected into tumors to minimize off-target side effects (Rizzo and Kehr, 2021). After that, injectable hydrogel would undergo sol-to-gel transformation in response to local biological milieu, forming a drug depot in injection site and then continuously releasing the encapsulated cargoes with the passive diffusion of molecules and the degradation of gel matrix (Dimatteo et al., 2018; Pertici et al., 2019). Since solid tumor has high interstitial pressure, the *in-situ* formed hydrogel scaffold could effectively prevent intra-tumoral drugs against being efflux from tumor tissues (Baumann et al., 2009; Deng et al., 2019).

The tumor killing effect of ICB therapy heavily depends on the abundance of CTLs in tumor tissues (HaanenConverting, 2017; Frederico et al., 2021; Zhang et al., 2022). Low tumor infiltration of CTLs is the main reason for the low response rate of ICIs (Balermpas et al., 2014; Stanton and Disis, 2016; Paijens et al., 2021). Some studies have combined ICB immunotherapy with chemotherapy, radiotherapy and other therapies that can induce immunogenic cell death (ICD) (Galluzzi et al., 2020; Xiang et al., 2022). ICD triggers the release of damage-associated molecule patterns (DAMPs) from tumor cells, such as calreticulin (CRT), adenosine triphosphate (ATP), and high mobility group protein 1 (HMGB1), facilitating the recruitment and activation of tumor-specific CTLs. Therefore, a combination with ICD inducers may be a powerful approach to improve statin-mediated ICB therapy. Collectively, we designed an *in-situ* formed codelivery platform to achieve potent ICB mediated by small molecule drugs. Atorvastatin (ATO) and cyclophosphamide (CTX) were co-loaded into an injectable *in situ* formed hydrogel. We hypothesized that the blockade efficiency of ATO could be amplified by both hydrogel-enhanced drug retention and CTX-mediated ICD induction, exhibiting a coordinated effect to inhibit tumor progression.

## 2 Materials and methods

### 2.1 Materials

Atorvastatin (ATO, catalog number: BD18106) and cyclophosphamide (CTX, catalog number: BD122996) were purchased from Bidepharm (China). Pluronic F127 was purchased from Sigma-Aldrich (United States). ATP Assay Kit (catalog number: S0026) was purchased from Beyotime biotechnology (China). Anti-CD16/32 (catalog number: 65080-1-L), anti-CD3-FITC (catalog number: FITC-65077), and anti-CD8a-APC (catalog number: APC-65069) were purchased from Proteintech (China). Anti-CD274 (PD-L1)-PE (catalog number: 124308) was purchased from Biolegend (United States). Anti-calreticulin primary antibody (catalog number: ab271865) was purchased from Abcam (United States). HMGB1 rabbit monoclonal antibody (catalog number: AG2167), *β*-actin mouse monoclonal antibody (catalog number: AF0003), Alexa Fluor 488-labeled goat anti-rabbit IgG (H + L) (catalog number: A0423), HRP-conjugated goat anti-mouse IgG (H + L) (catalog number: A0216), and HRP-conjugated goat anti-rabbit IgG (H + L) (catalog number: A0208) were purchased from Beyotime biotechnology (China).

### 2.2 Cells and animals

CT26 murine colon tumor cells were purchased from Chinese Academy of Science Cell bank (Shanghai, China) and incubated at 37°C in RPMI-1640 medium (Gibco) added with 10% fetal bovine serum (FBS) and 1% mixture of antibiotics. BALB/c mice were supplied by Dossy Experimental Animals Co., LTD. (Chengdu, China). Animal studies were conducted on the basis of the guidelines of Animal Ethics Committee of First People’s Hospital of Shuangliu District.

### 2.3 *In vivo* tumor retention

5 × 10^5−ΔΔCT^26 colon cells were subcutaneously injected into the right flank of BALB/c mice (6–8 weeks old). When the tumor volume increased to 300–500 mm^3^, 50 μL of free Rhodamine B (Rho B) or hydrogel loaded with Rhodamine B (Rho B@Gel) were injected into tumor tissues (Rho B dose, 5 μg/kg) (Liu et al., 2020; Hu et al., 2021). 24 h after injection, tumors were collected and captured by IVIS living image system.

### 2.4 Drug release profile

1 mL of Rho B and Rho B@Gel (Rho B, 10 μg/mL) were transferred into a dialysis tube (MWCO = 8–14 kDa) and then immersed in 40 mL PBS (pH 7.4) at 37°C avoiding light. At the determined time intervals, 1 mL buffer solution was collected and same amount of fresh phosphate buffer was supplemented. The obtained sample solution was analyzed using microplate reader to detect the release behavior of loaded cargoes.

### 2.5 PD-L1 expression

CT26 cells were treated with ATO (10 μg/mL), CTX (5 μg/mL) or their combination (10 μg/mL ATO + 5 μg/mL CTX) for 24 h. Cells were harvest and blocked with 5% goat serum at 4°C for 1 h. Then cells were stained with anti-CD274 (PD-L1) antibody (1:300) for another 1 h, followed by flow cytometry analysis.

### 2.6 Cytotoxicity evaluation

3-(4,5-dimethylthiazol-2-yl)-2,5-diphenyltetrazolium bromide (MTT) was employed to determine the cell viability. CT26 cells were treated with a serious concentration of ATO, CTX or their combination for 24 h. After that, 20 μL MTT solution (5 mg/mL) was added and incubated for another 4 h. Then, supernatant was replaced with 200 μL DMSO to dissolve the formazan and cell viability was measure by the absorbance at 570 nm.

### 2.7 CRT exposure, ATP secretion, and HMGB1 release

For qualitative study of CRT exposure, cells were mounted onto slides and treated with ATO (10 μg/mL), CTX (5 μg/mL) or their combination (10 μg/mL ATO+5 μg/mL CTX) for 12 h. Cells were incubated with calreticulin primary antibody (1:400) and Alexa Fluor 488-conjugated secondary antibody (1:1,000). The slides were observed by confocal laser scanning microscopy (CLSM). For quantification, cells were treated and stained as mentioned above, followed by flow cytometry analysis. To study extracellular ATP secretion and HMGB1 release, cells were treated with ATO (10 μg/mL), CTX (5 μg/mL) or their combination (10 μg/mL ATO + 5 μg/mL CTX) for 24 h. The supernatant was collected. ATP concentrations were analyzed by the standard protocol as reported before (Song et al., 2018; Chen et al., 2021). HMGB1 were assayed by Western blot.

### 2.8 *In vivo* antitumor study

5 × 10^5−ΔΔCT^ CT26 cells were subcutaneously injected to the right flank of mice to establish xenograft colon tumor model. When the tumor volume reached about 100 mm^3^ (day 7), 50 μL of saline, free ATO solution (50 mg/kg), free CTX solution (25 mg/kg), free ATO + CTX solution (50 mg/kg ATO +25 mg/kg CTX), or hydrogel containing ATO (ATO@Gel, 50 mg/kg), CTX (CTX@Gel, 25 mg/kg), or their combination (ATO + CTX@Gel, 50 mg/kg ATO +25 mg/kg CTX) was injected into the tumor using BD Ultra-Fine insulin syringe (29 G), respectively. Tumor surface with intact skin were chosen as the injection site. The tumor volume was computed according to the formula: (length×width^2^)/2. At the end point of experiment, mice were sacrificed. Subcutaneous tumors were separated for further use. The blood, heart, liver, spleen, lung and kidney were collected for safety evaluation.

### 2.9 Flow cytometry analysis of tumor-infiltrated T lymphocytes

Tumors in *in vivo* antitumor effect experiment were ground into single cell suspension. Then cells were blocked with anti-CD16/32 antibody (1:300) and stained with various antibodies against cell surface markers as reported before. CD3^+^CD8^+^ positive T cells were marked to reveal the provocation of antitumor immune response.

### 2.10 Safety evaluation

Body weight of mice in all groups were recorded during *in vivo* antitumor effect experiment. Serum was separated from bloods collected in *in vivo* antitumor experiment and the contents of aspartate transaminase (AST) and alanine aminotransferase (AST) were measured by full-automatic blood biochemical analyzer (cobas c311, Roche). Major organs were fixed, embedded in paraffin, and sectioned. After hematoxylin-eosin (H&E) stating, the morphologies of tissues were observed.

### 2.11 Statistical analysis

Data are presented as means ± SD (*n* ≥ 3). Two tailed unpaired Student’s *t*-test and one-way ANOVA was used to verify the significance of the results with Graphpad Prism 8.0.2 software.

## 3 Results and discussion

### 3.1 *In-situ* formed thermosensitive hydrogel improved the tumor retention of administrated small molecules

**FIGURE 1:**
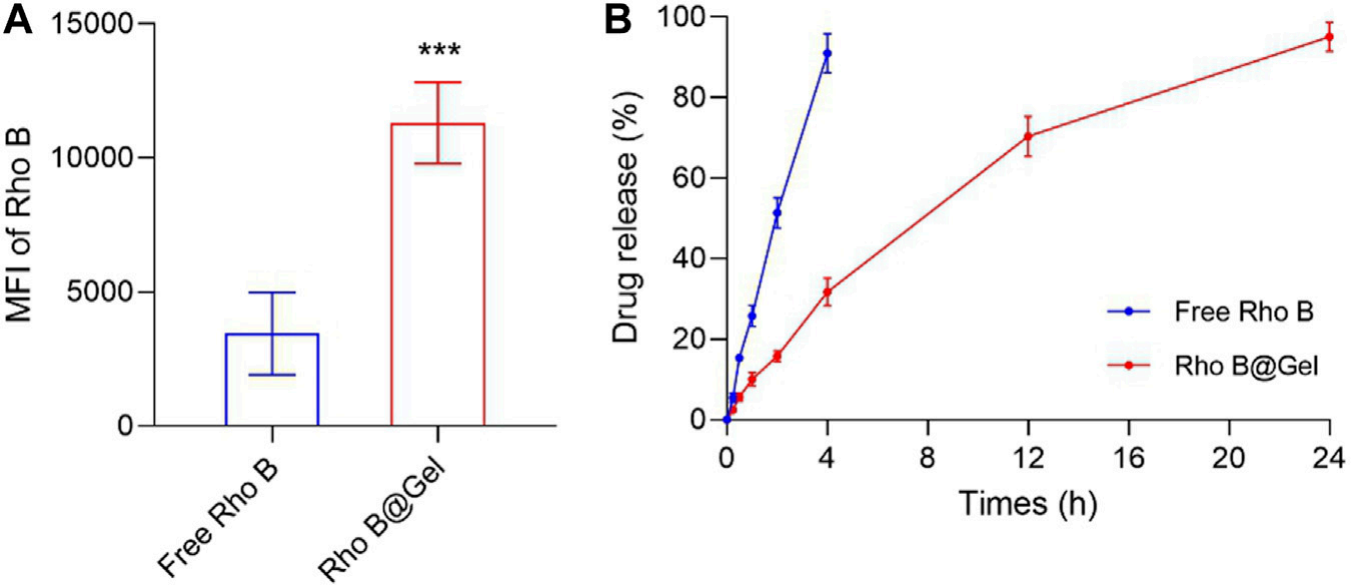
Pluronic F127 hydrogel improved drug retention in tumors. **(A)** Signals of Rho B in CT26 tumor tissues 24 h post intra-tumoral injection of 50 μL Free Rho B or Rho B@Gel (Rho B dose, 5 μg/kg) (*n* = 3). **(B)** Release profile of Free Rho B and Rho B@Gel (*n* = 3). Data was presented as mean ± SD. ***p < 0.001 versus Free Rho B group, Student’s *t*-test.

Among plenty of hydrogel matrix materials, we choose Pluronic F127 to construct *in-situ* drug reservoirs due to the following reasons. First, Pluronic F127 is a tri-block polymer composed of polyethylene and polypropyleneglycol. This linear polymer self-assembled into micelle at low concentration. Once at high concentration (>20%, w/v), the micelles would cross-link to each other, forming an immobile hydrogel scaffold with the increase of temperature (Gioffredi et al., 2016). The gelation temperature of Pluronic F127 solution is above 35°C, which is very suitable for *in vivo* application (Liu et al., 2007). Second, Pluronic F127 has been approved as an injectable drug excipient. After been injected into bodies, it can be gradually and completely degraded without generating any toxic products (Shachaf et al., 2010). Lastly, water-soluble drugs can be easily loaded into Pluronic F127 hydrogel by simply mixing Pluronic F127 solution and drug-containing solution (Ganguly et al., 2020). We hypothesized that the intra-tumoral injected F127 Gel would constrain drugs in tumor for a long time, thus increasing the accessibility of intra-tumoral drugs to tumor cells. Rhodamine B (Rho B), a hydrophilic dye, was introduced to visualize small molecules dissolved inside hydrogel. To demonstrated the enhanced retention of drug-loaded hydrogel in tumors, BALB/c mice with subcutaneous CT26 colon tumors were intratumorally injected with free Rho B and hydrogel loaded with Rho B (Rho B@Gel). After 24 h treatment, tumors were harvested. As shown in Figure 1A, tumor accumulation of Rho B for Rho B@Gel group is 3.27-fold higher than free Rho B group, validating the superiority of hydrogel scaffold in prolonging the retention of encapsulated drugs in tumors. The sustained drug release behavior of Rho B@Gel was shown in Figure 1B. Free Rho B quickly diffused out from dialysis tube. While the diffusion of Rho B was significantly retarded by Rho B@Gel, exhibiting a continuous release behavior in 24 h.

### 3.2 ATO downregulated PD-L1 expression and sensitized chemotherapy-induced ICD in colon tumor cells

**FIGURE 2:**
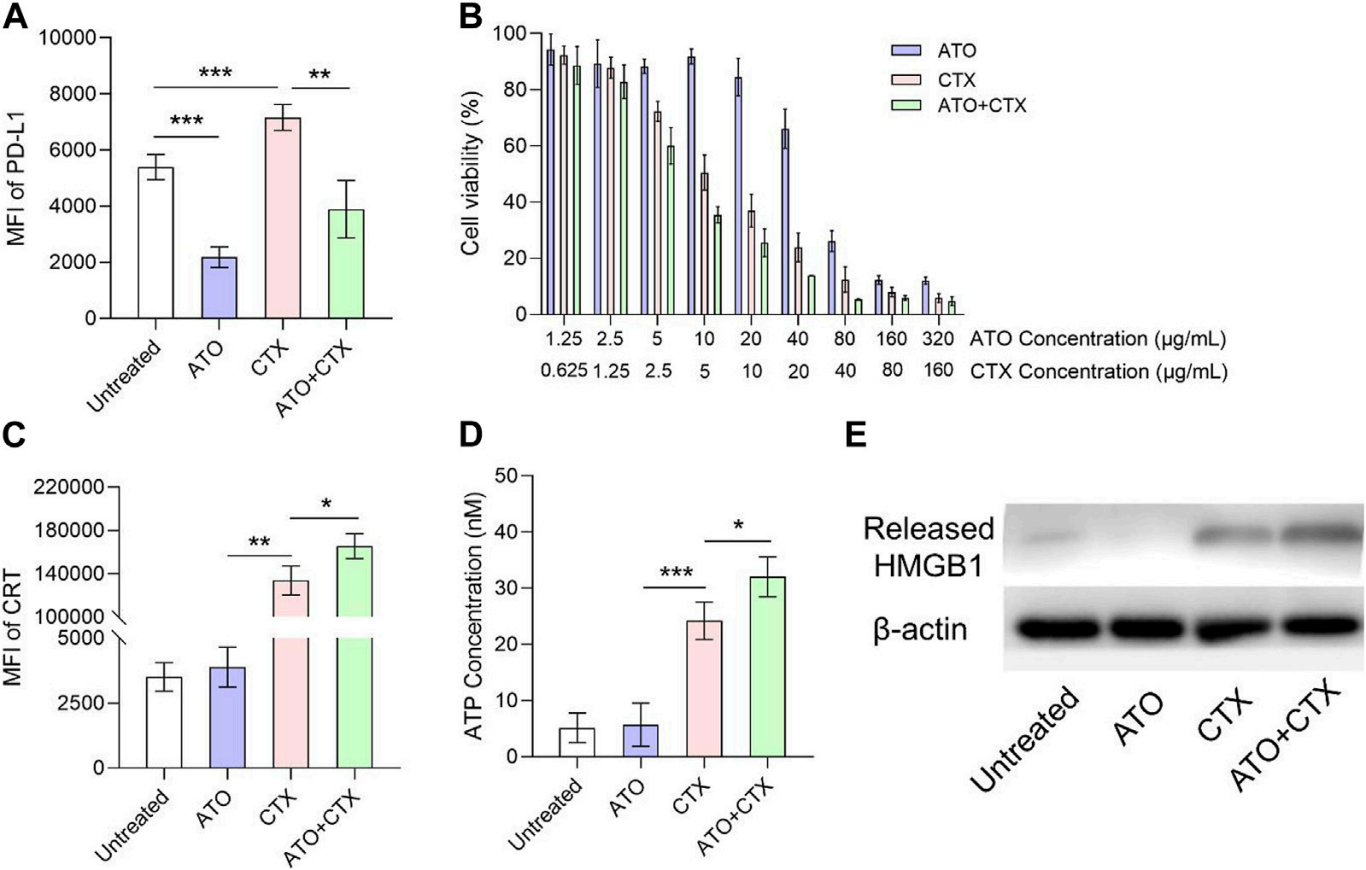
ATO downregulated PD-L1 expression and sensitized CTX-induced ICD in colon tumor cells after different treatments. **(A)** Flow cytometry analysis of PD-L1 on the surface of CT26 cells after treatments with ATO, CTX and their combination for 24 h (*n* = 3). **(B)** Viability of CT26 tumor cells after treated with a series of concentrations of ATO, CTX and their combination for 24 h (*n* = 3). **(C)** Flow cytometry analysis of surface CRT exposure on CT26 cells after treatments with ATO, CTX and their combination for 24 h (*n* = 3). **(D)** ATP concentrations in the supernatant of CT26 cells after treatments with ATO, CTX and their combination for 24 h (*n* = 3). **(E)** Western blot assay of the HMGB1 released from CT26 cells after treatments with ATO, CTX and their combination for 24 h *β*-Actin served as control. Data was presented as mean ± SD. *p < 0.05, **p < 0.01, ***p < 0.001, one-way ANOVA with Tukey’s multiple comparison test.

According to previous studies, PD-L1 is widely overexpressed on CT26 cells, which is very appropriate for evaluating the ICB efficiency of statins. ATO is one of hydrophilic statin drugs and its PD-L1 inhibition function has been validated in triple-negative breast cancer cells (Choe et al., 2022). The expression level of PD-L1 on CT26 cell surface upon ATO treatment was about 40% of untreated group (Figure 2A), indicating that ATO suppressed PD-L1 expression on colon cancers. It should be noted that CTX upregulated PD-L1 on CT26 cells (Figure 2A), which was in line with other chemotherapeutic drugs (Liu et al., 2022a; Liu et al., 2022b). Compared with single treatment of CTX, treatment of CTX + ATO had a lower PD-L1 expression (Figure 2A), indicating that chemotherapy-induced PD-L1 upregulation could be reversed by statins.

Results of cell viability assay showed that ATO had very little cytotoxicity on CT26 cells at its PD-L1-lowing concentration (10 μg/mL) (Figure 2B), highlighting the requirement of a combination with cytotoxic agents. CTX effectively killed CT26 cells (about 50%) at the concentration of 5 μg/mL, and the combination with ATO slightly improved the cytotoxicity of CTX (Figure 2B). Chemotherapeutic drug CTX was expected to improve ICB therapy by inducing ICD. CRT exposure, ATP secretion, and HMGB1 release are three key hallmarks in ICD process, and the effects of CTX on these hallmarks were tested at 5 μg/mL. As shown in Supplementary Figure S1, untreated CT26 cells showed negligible surface CRT expression, the fluorescence of CRT is mainly distributed in cytoplasm. In contrast, tumor cells treated with CTX exhibited a significant CRT translocation from cytosol to the surface of CT26 cells (Supplementary Figure S1). Similar results were also acquired by flow cytometry (Figure 2C). A chemiluminescence method was utilized to detect the ATP concentration in culture medium after different treatments. As shown in Figure 2D, ATP was rarely detected in the supernatant of untreated cells, but the ATP concentration notably increased after CTX treatment. Extracellular HMGB1 release was also investigated. CTX significantly triggered HMGB1 release as compared with untreated cells (Figure 2E). These results confirmed that CTX effectively induced ICD on CT26 colon tumor cells. Interestingly, we found that the degree of CTX-induced ICD could be amplified by ATO, even though ATO itself was unable to induce ICD. This was demonstrated in Figures 2C–E.

### 3.3 Hydrogel-based drug delivery achieved potent tumor immunotherapy via a synergism of checkpoint blockade and T cell activation

**FIGURE 3:**
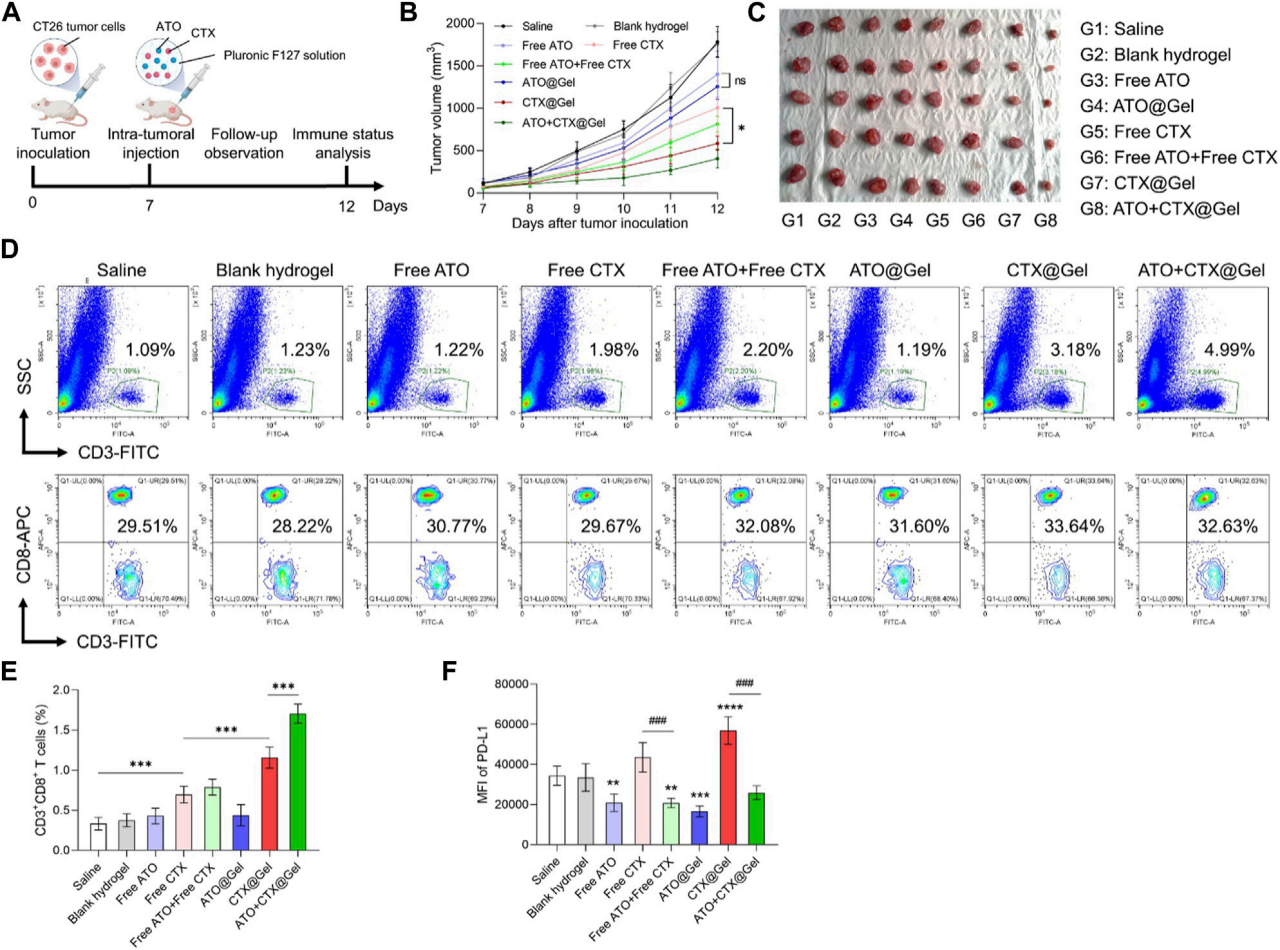
ATO + CTX@Gel achieved potent tumor chemo-immunotherapy. **(A)** Time schedule for *in vivo* antitumor experiment. **(B)** Tumor growth curves and **(C)** images of CT26 tumors at the endpoint after different treatments (*n* = 5). Ns, not significant, *p < 0.05. **(D)** Representative flow cytometry plots and **(E)** quantitative analysis of the percentage of CD3^+^ T cells and CD3^+^CD8^+^ T cells in CT26 tumors after different treatments (*n* = 5). ***p < 0.001. **(F)** Flow cytometry analysis of PD-L1 expression on CT26 tumors after different treatments (*n* = 5). **p < 0.01, ***p < 0.001, ****p < 0.0001 versus Saline group, *###p* < 0.001. Data was presented as mean ± SD. Statistical analysis were performed via Student’s *t*-test and one-way ANOVA with Tukey’s multiple comparison test.

To evaluate the *in vivo* potency of our platform, BALB/c mice bearing CT26 colon tumor xenografts were intratumorally injected with saline or different formulations, respectively (Figure 3A). Because of the fast proliferation of CT26 cells, the tumor volumes of Saline group increased drastically (Figure 3B). The antitumor effect of free ATO and ATO@Gel is very weak because ATO is not a cytotoxic drug that directly inhibit cell growth (Figure 3B). In comparison, CTX exhibited noticeable tumor suppression as a chemotherapeutics. The anti-tumor effect of CTX was significantly improved after being loaded into hydrogel (*p* < 0.05, Free CTX group versus CTX@Gel group) (Figure 3B), indicating that prolonged drug retention in tumor by hydrogel might be benefit to the tumor suppressive effect of chemotherapeutics. More importantly, the combination of ATO and CTX in hydrogel achieved the best tumor regression (Figures 3B,C), implying a potential coordination between these two drugs.

To disclose underlying mechanisms behind superior tumor inhibition of ATO + CTX@Gel, immune status in tumors was investigated. Cancer cells succumbing to ICD inducers like CTX can be converted into vaccine that stimulate antitumor immune response. As shown in Figures 3D,E, compared with Saline group, Free CTX improved the tumor infiltration of CD3^+^CD8^+^ T lymphocytes, indicating the activation of antitumor immunity. In comparation, blank hydrogel, Free ATO and ATO@Gel did not change the population of intra-tumoral CD3^+^CD8^+^ T cells. The efficacy of CTX on T cell activation could be mildly improved by hydrogel encapsulation (*p* < 0.001, Free CTX group versus CTX@Gel group), which demonstrating the indispensable role of drug retention in tumor immunotherapy. It should be noted that ATO + CTX@Gel clearly modified the flow cytometry plot (SSC plus CD3) in comparison to those obtained with free drugs. This is because ATO encapsulated in hydrogel could sustainedly release and downregulate PD-L1 in tumor tissue, which decreased the immunosuppressive barriers against T cell recruitment by CTX. As a result of continuous T cell recruitment and reactivation, ATO + CTX@Gel induced the highest portion of CD3^+^ T cells infiltrated into tumors.

The reduction of PD-L1 expression on tumor cell surface unleashed the “brake” of antitumor immunity and circumvented immune escape, allowing CTLs to target malignant tumor cells. To further reveal the superiority of ATO + CTX@Gel over CTX@Gel in tumor inhibition (Figures 3B,C), PD-L1 expression in tumors tissues was investigated after treatments. As shown in Figure 3F, tumors treated with free ATO or ATO-containing hydrogels showed a lower PD-L1 expression level than Saline group, and even reversed the PD-L1 upregulation after CTX-mediated tumor killing. These results firmly demonstrated that the synchronization of causing CTLs-based antitumor immunity and reducing PD-L1 mediated immune escape is very important for tumor suppression.

### 3.4 Safety and biocompatibility evaluation

**FIGURE 4:**
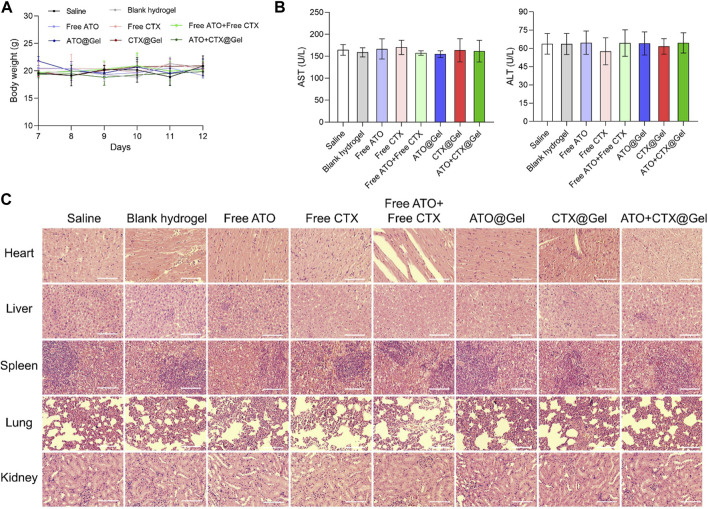
Safety and biocompatibility evaluation. **(A)** Changes in body weight and **(B)** levels of serum AST and AST after different treatments (*n* = 3). **(C)** Hematoxylin and eosin (H&E) staining of the main organs of mice after different treatments. Scale bar = 50 μm. Data was presented as mean ± SD.

As compared with systemic injection, intra-tumoral chemo-immunotherapy has the advantage of lower drug dose and decreased off-target effects. In order to evaluate the potential of further application, the safety and biocompatibility of our regimes are comprehensively analyzed. During the treatment, no body weight loss was observed in all groups (Figure 4A). After action, small molecule drugs are metabolized and degraded in the liver. The level of aspartate transaminase (AST) and alanine aminotransferase (AST) did no differ between the groups treated with saline and various formulations (Figure 4B), indicating that all preparations had no obvious hepatotoxicity. At the endpoint of various treatments, major organs of all groups including heart, liver, spleen, lung and kidney were collected for histological staining. As shown in Figure 4C, no pyknosis or edema of liver nuclei was found in the liver tissue sections of all groups. In addition, no obvious abnormality was observed in the myocardium, spleen and glomerulus between different groups, demonstrating good *in vivo* safety of our therapies.

## 4 Conclusion

Although many small molecule drugs and their combination with immune checkpoint blockade antibodies have proved clinical efficacy against solid tumors, one of the major limitations of these therapies is their rapid diffusion from the target tissue (Zhuang et al., 2019; Liu et al., 2022a; Liu et al., 2022b). The incorporation of ICD-inducing chemotherapeutics and PD-L1-inhibiting drugs into a hydrogel drug delivery system is sufficient to overcome this difficulty and realize satisfactory tumor inhibition. In this study, we proved that the combination of ATO and CTX in hydrogel could activate anticancer immune response through PD-L1 blockade and ICD induction. The hydrogel significantly improved the tumor retention and tumor regression ability of small molecule drugs. With this advantage, ATO + CTX@Gel suppress tumor growth in CT26 colon cancer xenograft models. This approach can make up for the lack of tumor targeting and retention of small molecule drugs, generating robust ICD and overcome the immune escape of tumor cells. Another advantage of this platform is to avoid space-time dislocation of different drugs in combination therapy. Different drugs can be loaded into one hydrogel, which can synchronize the required drug regimens and provide a potential method for tumor chemo-immunotherapy.

## Data Availability

The original contributions presented in the study are included in the article/Supplementary Material, further inquiries can be directed to the corresponding authors.
