# Supplementary material for: In-situ formed thermosensitive hydrogel amplifies statin-mediated immune checkpoint blockade for coordinated tumor chemo-immunotherapy
Source: Front Pharmacol. 2023 May 9;14:1154392. doi: 10.3389/fphar.2023.1154392 (PMC10204804; doi:10.3389/fphar.2023.1154392)
Supplement: Supplementary file 1 [file DataSheet1.docx]

Supplementary Material

In-situ formed thermosensitive hydrogel amplifies statin-mediated immune checkpoint blockade for coordinated tumor chemo-immunotherapy

Zefan Liu^1^*, Xin Kang^1^*

Department of General Surgery, First People’s Hospital of Shuangliu District, Chengdu, China

*** Correspondence:**

Xin Kang: kangxin20220726@163.com

Zefan Liu: 77868808@qq.com

# Supplementary Figures


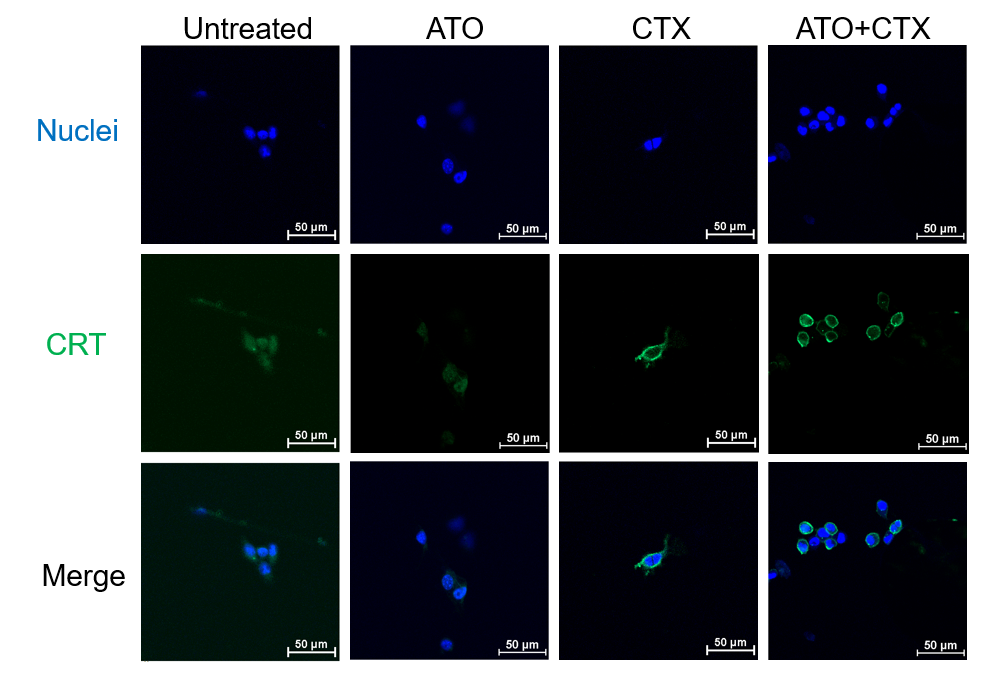


**Figure S1.** The extracellular CRT exposure of CT26 cells after treatments with free atorvastatin (ATO), cyclophosphamide (CTX) or both in combination. Surface CRT was conjugated with rabbit polyclonal

CRT primary antibody and further incubated with Alexa Fluor 488 labeled secondary antibody at 4 °C. Blue: Nuclei, Green: CRT. Scar bar: 50 μm.


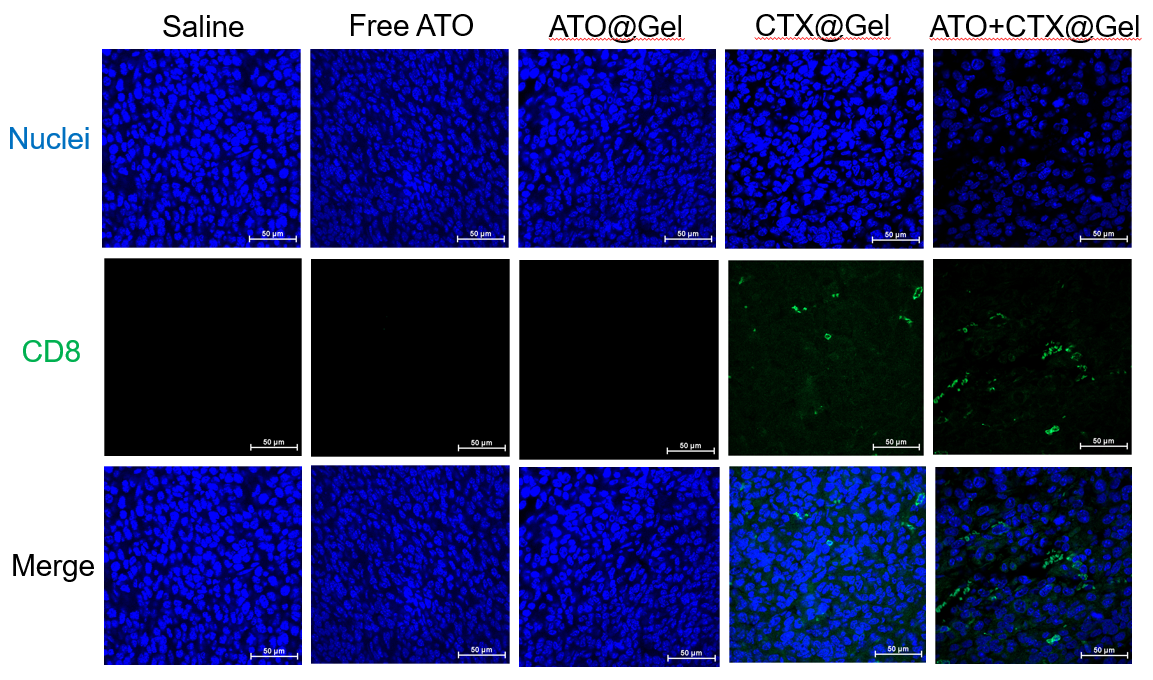


**Figure S2.** Immunofluorescence staining of the infiltration of CD8+ T lymphocytes in tumors collected in saline, free ATO, ATO@Gel, CTX@Gel, ATO+CTX@Gel treated mice. Blue: Nuclei, Green: CD8^+^ T cells. Scar bar: 50 μm.
